# Experimental Study on Damage Monitoring of FRP Plate Using FBG Sensors

**DOI:** 10.3390/mi16060649

**Published:** 2025-05-29

**Authors:** Zhe Zhang, Tongchun Qin, Yuping Bao, Ronggui Liu, Jianping He

**Affiliations:** School of Civil Engineering, Nantong Institute of Technology, Nantong 226000, China; zhangzhe_nt@126.com (Z.Z.);

**Keywords:** FRP plate, fiber Bragg grating, damage identification, impulse load, continuous sinusoidal vibration load

## Abstract

With the widespread application of FRP (Fiber Reinforced Plastics) materials in fields such as wind turbine blades and ships, the safety performance of these materials during their service life has garnered signification attention. This study employs the fiber Bragg grating (FBG) sensor to monitor damage of the FRP materials. An FRP plate embedded with six FBGs was fabricated, and different degrees of damage were induced in the FRP plate. The six FBGs measured the damage information of the FRP plate under impulse and continuous sinusoidal vibration loads. The results demonstrate that both the strain information and the frequency shift information measured by the FBG sensors can effectively and sensitively identify damage in the FRP plate.

## 1. Introduction

FRP composite materials are increasingly utilized in civil engineering, shipbuilding, wind turbines, and aerospace due to their lightweight, high-strength, and corrosion-resistant properties. However, FRP components often operate in harsh environments under long-term alternating loads, leading to inevitable damage such as matrix cracking, interface debonding, and fiber fracture. While some fibers may continue to bear loads, delaying catastrophic failure, it is crucial to monitor the mechanical properties of FRP components during their service life to assess their safety and longevity. This has become a critical research area in the application of FRP composites.

Current research methods for evaluating FRP performance include finite element simulation and data-driven performance assessment. Finite element analysis (FEA) accurately predicts the response characteristics of FRP structures and facilitates damage simulation, making it a vital tool for the design and maintenance of FRP components. For instance, Bilotta et al. experimentally tested glass FRP-RC (Fiber Reinforced Plastics–Reinforced Concrete) panels under ISO 834 standard fire conditions using FEA [1]. Kharghani et al. investigated the behavior of hybrid composite-steel balcony overhangs on ships through numerical and experimental approaches [2]. To truly obtain the performance of FRP composite structures during operation, strain sensors, vibration sensors, digital image correlation (DIC), and other means are directly or indirectly installed on FRP components or structures to obtain the corresponding structural deformation and vibration response characteristics [3,4,5,6,7]. Optical fiber sensing technologies, such as fiber Bragg grating (FBG) and distributed optical fiber sensing technologies including BOTDR (Brillouin optical time domain reflectometry) or OFDR (Optical Frequency Domain Reflectometry), are particularly advantageous due to their small size, resistance to electromagnetic interference, corrosion resistance, and long signal transmission distances, making them ideal for strain, vibration, pressure, and temperature measurements [8,9,10,11,12]. Chen et al. designed a sensor layout based on FBGs in series to measure the dynamic response of one CFRP antenna beam [13]. Shi et al. used FBG and distributed Brillouin optical fiber sensing technology to monitor the circumferential strains of steel pipes and CFRP confined seamless/cracked steel pipes in a hydrostatic testing [14].

Optical fiber or FBG are relatively thin and easily damaged by shear. To enhance the engineering characteristics of distributed optical fiber or FBG, Zhou et al. embedded optical fibers or FBGs into FRP composite materials. This encapsulation process not only protects the sensors but also ensures efficient strain transfer between the optical fibers and the FRP matrix [15]. Wang et al. further provided theoretical insights into strain transfer for FRP-encapsulated optical fiber sensors, guiding their optimal design [16,17]. Additionally, the small diameter of optical fibers or FBGs minimally affects the mechanical properties of FRP, allowing their integration into various FRP structures for intelligent monitoring [18,19,20,21].

Damage such as fiber breakage and delamination peeling within FRP have been hot and difficult research issues [22]. Yashiro et al. proposed one approach to predicting multiple damage states in composite laminates using embedded fiber Bragg grating (FBG) sensors [23]. FRP, as a protective structure for ship structures, is prone to discontinuous damage, such as cavities under external impacts. In order to study the damage of FBGs under vibration impacts on FRP structures, six FBGs were embedded into an FRP plate to monitor damage of the FRP plate under impulse and continuous sinusoidal vibration loads in this study. To obtain the damage information, four levels of damage of the FRP plate were artificially induced, and one impulse load was applied at the center of the FRP plate at each damage condition, and the corresponding damage identification was conducted using time and frequency domain analysis.

## 2. Sensing Principle of FBG

FBG is a passive device whose central wavelength varies linearly with strain and temperature, as expressed by:

(1) $$\Delta \lambda =C_{\varepsilon }\Delta \varepsilon +C_{T}\Delta T$$

where Δλ is the central wavelength variation of FBG; Δε,ΔT are the strain and temperature variations, respectively; and $C_{\varepsilon }(C_{\varepsilon }=1.2\;pm/\mu \varepsilon )$ and $C_{T}(C_{T}=10.8\;pm/{}^{\circ}C)$ are strain and temperature sensitivity coefficients, respectively [24,25].

**Figure 1:**
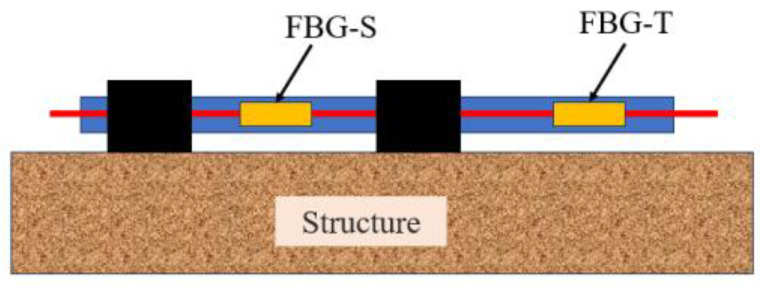
Sensor layout for temperature compensation method.

In practical applications, temperature fluctuations can introduce measurement errors in FBG-based sensors. To mitigate this, an FBG temperature sensor (FBG-T) is deployed alongside the FBG strain sensor (FBG-S) for temperature compensation, as shown in Figure 1. While the FBG-S senses both temperature and strain, as expressed in Equation (1), FBG-T only senses temperature, as expressed in Equation (2):

(2) $${\Delta \lambda }_{T}=C_{TT}\Delta T$$

Based on Equations (1) and (2), we have:

(3) $$\Delta \lambda =C_{\varepsilon }\Delta \varepsilon +{\Delta \lambda }_{T}C_{T}/C_{TT}$$

## 3. Damage Monitoring Test of FRP Plate Under Impulse Load

### 3.1. FBG Layout and Test Equipment

**Figure 2:**
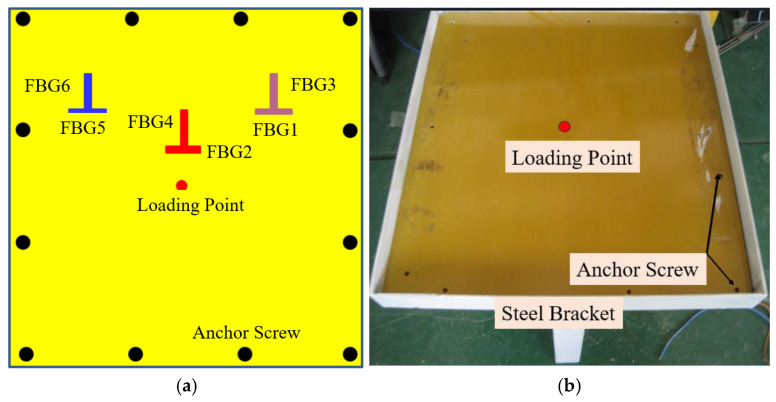
The FBG intelligent FRP plate. (**a**) FBG layout on the FRP plate; (**b**) Photo of FRP plate.

**Figure 3:**
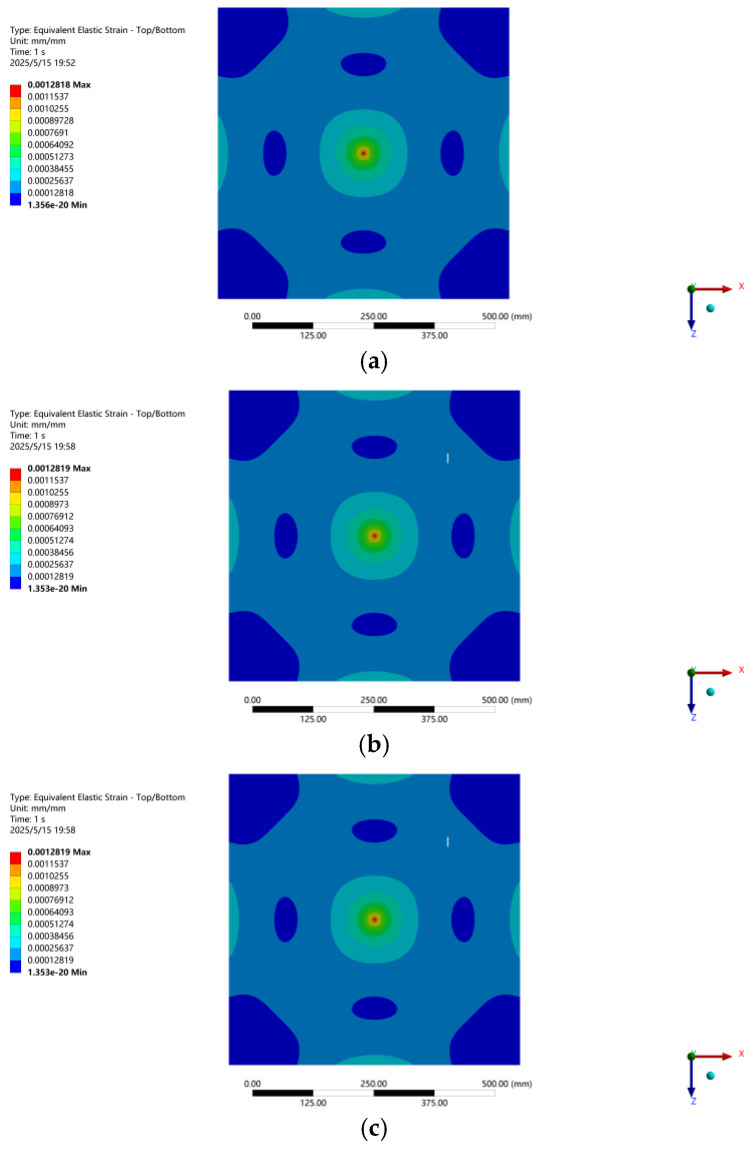

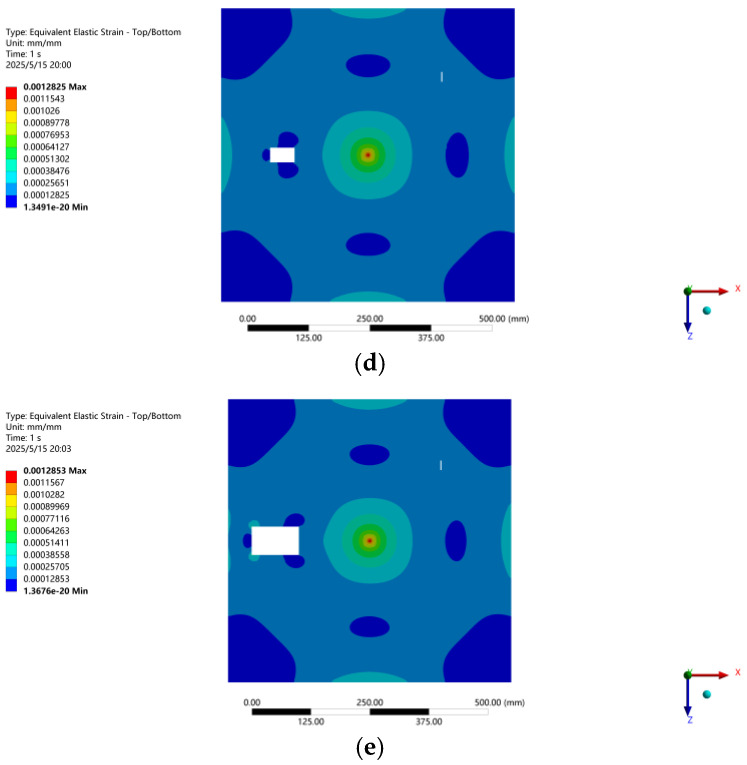
FEA result of FRP plate with different level damages. (**a**) No damage; (**b**) Damage-I; (**c**) Damage-II; (**d**) Damage-III; (**e**) Damage-IV.

Figure 2 is the picture of the glass FRP plate. The FRP plate is developed by the pultrusion molding process. The yarn content of the FRP plate is 62%, and the glass fiber filaments are laid in two layers. The side length of the FRP board is 600 mm, and the thickness is 2.16 mm. The fiber in the FRP board is glass fiber, and its module is 50 GPa. The FRP plate was screwed around the perimeter to the bracket. Figure 3 is the finite element analysis result of the FRP plate under 100 N. Figure 3a shows the finite element analysis of the FRP plate in the undamaged state, and Figure 3b–e show the finite element analysis of the FRP plate with a small crack (Damage-I), large crack (Damage-II), small damage (Damage-III), and larger damage (Damage-IV). For the small crack, the length and width of the cracks were 10 mm and 1 mm, respectively; for the larger crack, the length and width of the crack were 20 mm and 2 mm, respectively. While the small damage consists of the larger crack and one small rectangle hollow 50 mm long by 30 mm wide, the larger damage consists of the larger crack and one small rectangle hollow 100 mm long by 60 mm wide. The crack location is 150 mm from the center of the FRP plate. In the FEA of the no damage state, the SHELL181 shell unit is used for modeling, and the finite element model was modeled with 14,641 nodes and 14,400 elements. Theoretically, damage will affect the stress distribution of the FRP plate to some degree, and the closer to the location of the damage, the greater the effect. It also can be seen that the maximum strain location of the FRP plate is the location of the loading point, and the strain at the loading point and the crack location in the undamaged state are 392 με and 154 με, respectively; the stresses at the loading point and the crack location in the larger damage state are 395 με and 123 με, respectively. Based on the FEM results, the position of the maximum strain is the loading point, and in the vicinity of the rectangular crack, the strain values are progressively smaller. We chose the positions of 100 mm and 150 mm from the loading point to lay FBG sensors (named FBG1~FBG6), and two FBG sensors were laid orthogonally at each position. The initial wavelength of the FBGs is listed in Table 1. FBGs are fabricated using femtosecond technology on Corning single-mode fibers with greater than 95% reflectivity. In practice, the FBGs can be implanted directly into the FRP sheet as a fiber bundle during the FRP molding process. In this test, considering that the FBGs are laid on the FRP plate along the horizontal and vertical directions, we carved a 0.5 mm fine groove on the molded FRP plate, and the FBGs were laid with epoxy resin adhesive. The test was conducted indoors, and the indoor temperature was basically kept constant during the test, so no temperature compensation sensor was deployed for this test.

**Figure 4:**
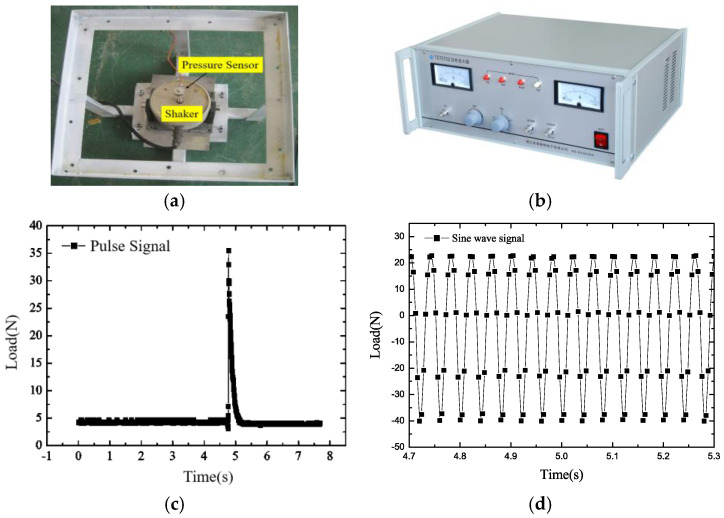
Shaker system: shaker and pulse load. (**a**) Shaker; (**b**) Power amplifier; (**c**) Impulse load; (**d**) Continuous sinusoidal vibration load.

Figure 4 shows the shaker system, which is mainly used to generate impulse dynamic loads and continuous sinusoidal vibration loads on the FRP plate.

### 3.2. Damage Analysis Under Dynamic Load

**Figure 5:**
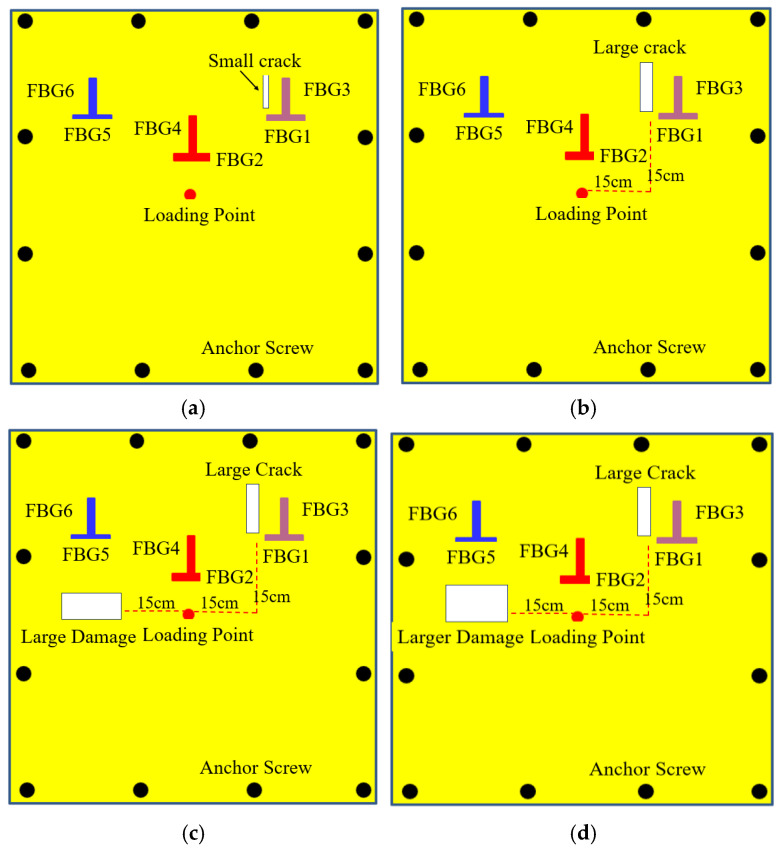
Four damage conditions of the FRP plate. (**a**) Small crack; (**b**) Large crack; (**c**) Large damage; (**d**) Larger damage.

**Figure 6:**
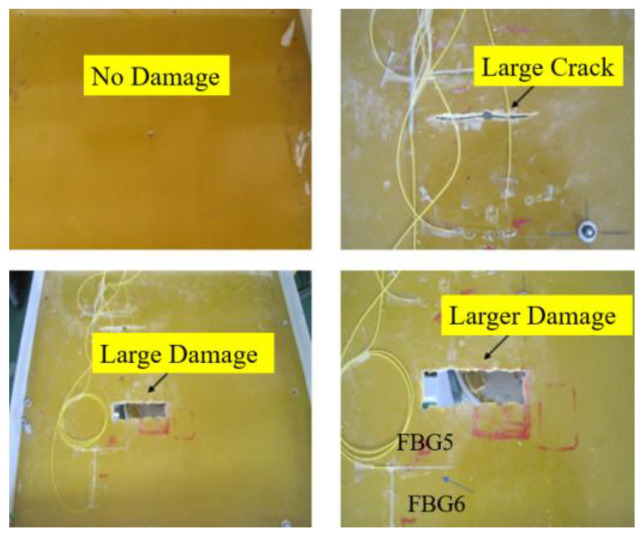
Picture of the damage condition of the FRP plate.

**Figure 7:**
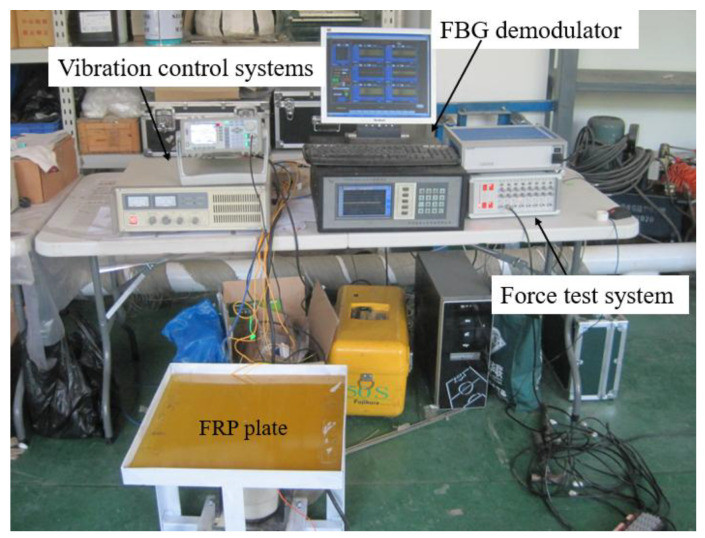
Test equipment system.

To obtain the strain information of the FRP plate under dynamic loads, four levels of damage close to the FBG sensors were induced in the FRP plate: small crack, large crack, large damage, and larger damage. The damage locations and dimensions are illustrated in Figure 5. The length and width of the small crack are 10 mm and 2 mm, respectively, and that of the large crack are 20 mm and 4 mm, respectively. Cracks are 15 cm away from the loading point both horizontally and vertically. The large damage includes one large crack and one rectangular-shaped injury, and the larger damage includes one large crack and one large rectangular-shaped injury. Figure 6 is the actual picture of the damage conditions of the FRP plate. A shaker system was used to apply impulse loads, and the strain responses were measured using an FBG demodulator (produced by Dalian Bo Ruixin Technology Co., Ltd., Dalian, China) with a sampling frequency of 250 Hz, an accuracy of 1.5 pm, and 32 channel numbers, as shown in Figure 7. In the test, the center of the FRP plate was bolted to the impact bar of the vibration device in order to obtain the force analysis of the FRP plate in the up and down directions, that is, the direction of the impulse load is alternating, named positive impulse load and negative impulse load, respectively. At the same time, the free oscillation of the FRP plate is small due to the restraining effect of the impact bar.

### 3.3. Data Analysis and Processing Under Pulse Load

**Figure 8:**
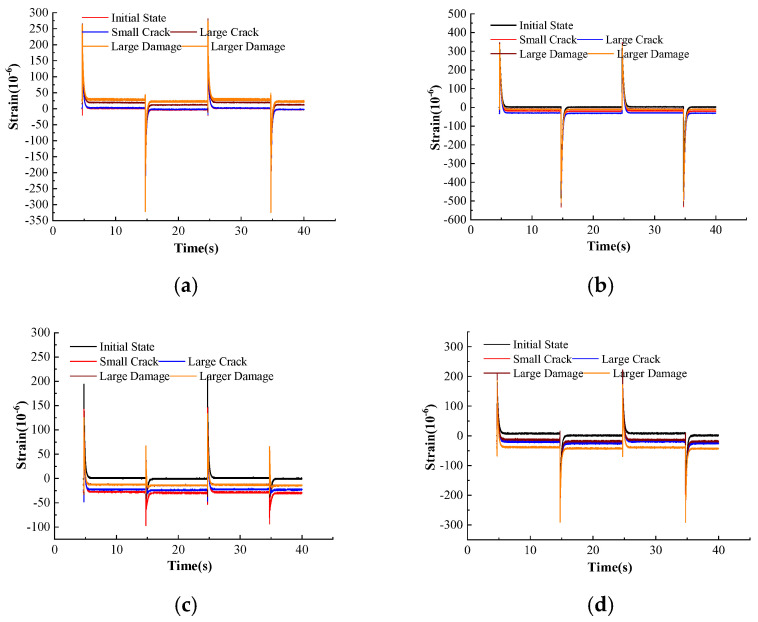

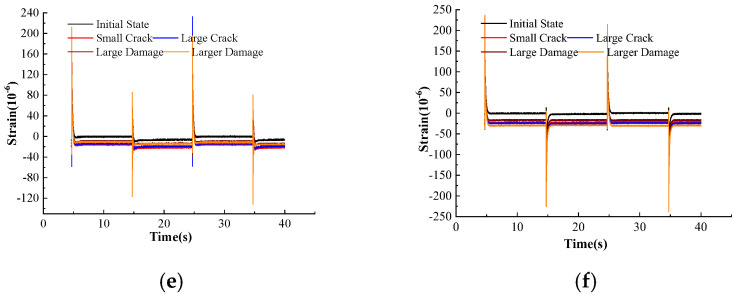
Strain values at different levels of damages. (**a**) FBG1; (**b**) FBG3; (**c**) FBG2; (**d**) FBG4; (**e**) FBG5; (**f**) FBG6.

During the pulse excitation test, the interval time between each pulse load is 10 s. In this time interval, the FRP plate can be free to damp the vibration until equilibrium after the impact. The strain data from FBG1 to FBG6 were recorded over 40 s, showing good repeatability across all damage levels, as shown in Figure 8. From the results, the damage of the FRP plate can be portrayed in two ways. The first way is the strain at various levels of damage vs. the strain in the undamaged state, and the second way is the comparison of the stabilization rates of FRP plates after impact loading. From Figure 8a, it can be seen that the maximum strain values measured by FBG1 are 187.35 με, 143.74 με, 205.27 με, 177.66 με, and 188.97 με at all levels of damage status, respectively, under the positive impulse load, while under the negative impulse load, the maximum strain values are −263.93 με, −124.83 με, −203.97 με, −206.73 με, and −179.28 με, respectively. Meanwhile, with the increase in damage, the residual strain in the steady state of the FRP plate after impact loading is gradually increasing under the positive impulse load. The residual strain in the nondestructive state is 0.625με, and the residual strains for the first to the fourth level of damage are 2.23 με, 14.51 με, 28.47 με, and 25.81 με, respectively. Under the negative pulse load, the residual strain in the nondestructive state is −2.3 με, and the residual strains for the first to the fourth level of damage are −1.64 με, 12.89 με, 24.22 με, and 22.58 με, respectively. The strain directions measured by FBG3 and FBG1 are perpendicular to each other, and it can be seen that the two maximum strain values in mutually perpendicular directions are not the same. Figure 8b shows the maximum strain values measured by FBG3. Under the positive impulse load, the maximum strain values are 318.92 με, 339.17 με, 278.66 με, 345.92 με, and 337.37 με, respectively, while the maximum strain values are −484.458 με, −532.86 με, −505.88 με, −526.11 με, and −508.13 με, respectively, under the negative pulse load. The residual strains are −6.23 με, −20.24 με, −32.05 με, −10.12 με, and −11.81 με at the different levels of damage.

Figure 8c shows the strain measured by FBG2. Compared with the nondestructive state, in the first level of damage (Damage-I) and second level of damage (Damage-II), the strain changes are about −26 με and −24 με; also, compared with the nondestructive state, in the third level of damage (Damage-III) and the fourth level of damage (Damage-IV), the stresses of the FRP plate are redistributed, and the strain changes are about −13.2 με and −13.3 με, and the vibration decay smooth time is faster than the 1st~2nd damage modes, but the maximum dynamic strain in the fourth level is about −180 με, and the vibration decay smoothing time is faster than that of the level 1~2 damage modes, and the maximum strain of level 4 damage is about −180 με. Figure 8d shows the strain measured by FBG4. Compared to the undamaged condition, the strain changes in the first four damage conditions are −17 με, −27 με, −17 με, and −35 με, respectively, with a maximum strain of about −100 με for Damage- IV. Figure 8e shows the strain measured by FBG5. Compared with the nondestructive state, the static strain variation at the FBG5 position is in the range of −7 to −13 με for all levels of damage conditions, and the maximum strain of the fourth level of damage is about 150 με. Figure 8f shows the strain measured by FBG6. Compared to the undamaged state, the static strain variation at the FBG6 position is in the range of −18 to −28 με for all levels of damage conditions, with a maximum strain of about 150 με for Damage-IV.

Based on the analysis of the strain measurements at each location, we can direct that since FBG4 and FBG6 are relatively far away from the damage location, the damage has the least effect on them, while FBG2 and FBG5 are closer to the damage, where strain redistribution has a greater effect. It is also known that the local strain at the crack tip may increase during crack extension, but the strain in the surrounding area may decrease due to stress redistribution based on the FEM, as shown in Figure 3e, and the strain measured by FBG2.

**Figure 9:**
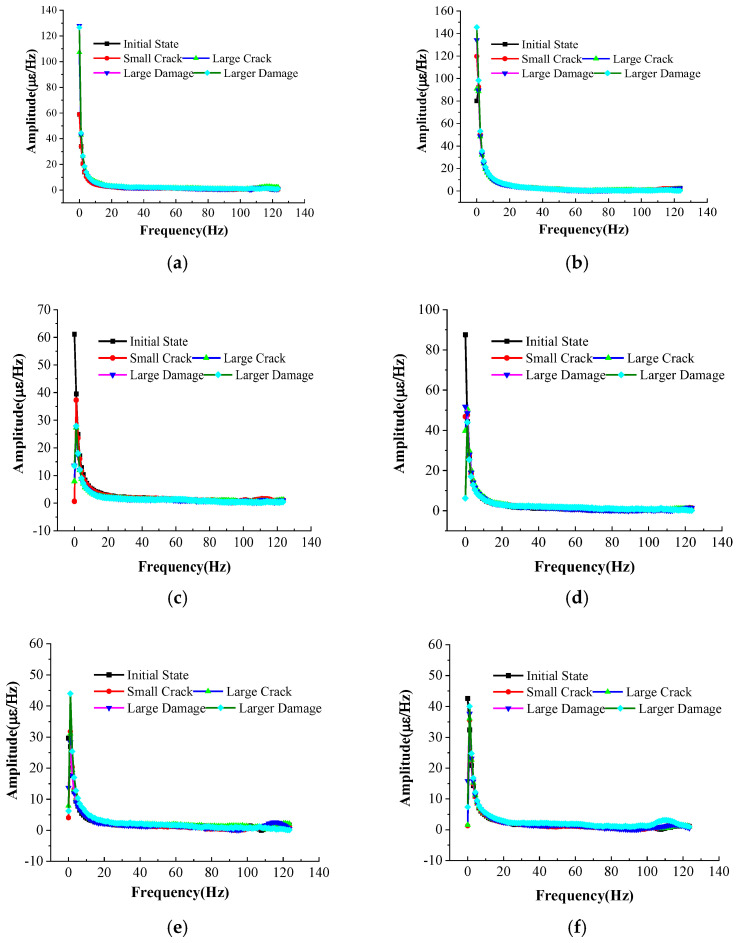
Amplitude–frequency relationship curve. (**a**) FBG1; (**b**) FBG3; (**c**) FBG2; (**d**) FBG4; (**e**) FBG5; (**f**) FBG6.

To characterize the frequency domain information of the FRP plate, Fourier transforms were performed on the strain data, as shown in Figure 9.

**Figure 10:**
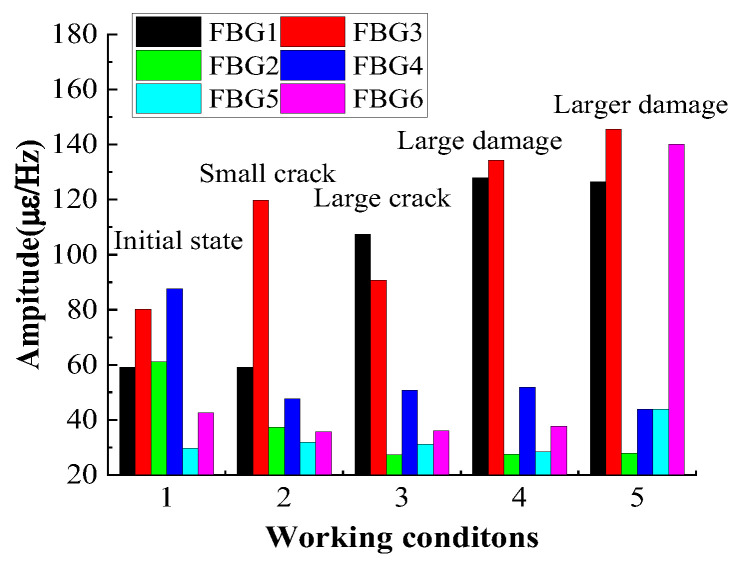
Maximum amplitude at each location correlates with damage status of FRP plate.

Figure 10 shows that the maximum magnitude at each location correlates with the damage status of the FRP plate. It can be seen that the maximum magnitudes are different for different damage states of the FRP plate. Comparing the two working conditions, large damage and larger damage, it shows that there is a large increment in the maximum amplitude at the FBG6 position of the FRP plate. Furthermore, the maximum magnitude measured by FBG2 decreases with the increasing degree of damage of the FRP plate, while the maximum magnitude measured by FBG4 is less affected by the level of damage. For FBG2, it is close to the larger damage (rectangular damage), so the constraint on the FRP plate at the location near FBG2 is reduced. Therefore, as the damage increases, the strain value measured by FBG2 becomes smaller. Comparison of the strain measurements and maximum magnitudes for FBG1 and FBG3, FBG2 and FBG4, and FBG5 and FBG6 shows that the direction of the FBG deployment leads to great variability in the measurements at the same location.

### 3.4. Data Analysis and Processing Under Continuous Sinusoidal Vibration Load

**Figure 11:**
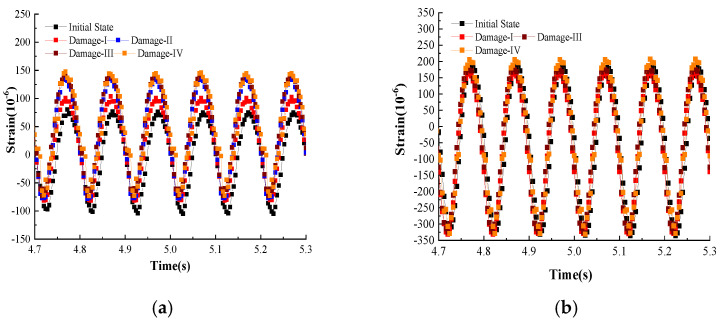

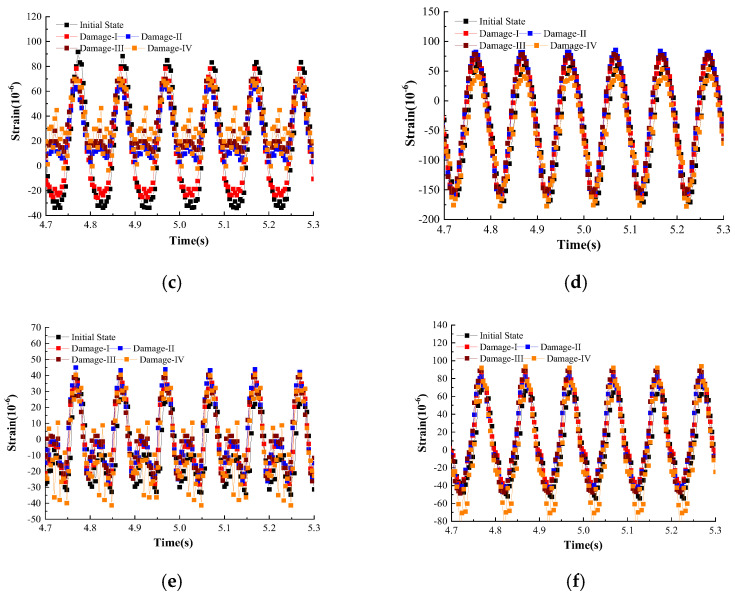
The strain values under continuous sinusoidal vibration load. (**a**) FBG1; (**b**) FBG3; (**c**) FBG2; (**d**) FBG4; (**e**) FBG5; (**f**) FBG6.

Figure 11 shows the strain values under the continuous sinusoidal vibration load measured by FBG1–FBG6. The vibration time is 0.6 s and contains five full sine waves. Again, it can be seen that the strain values measured by FBG2 and FBG5 increase with damage, and the strain curves behave in a more heterogeneous manner and are not sinusoidal. The main reason for this is that the third and fourth levels of damage are closer to FBG2 and FBG5, and the damage has a greater effect on the stresses in this region. The strain values at other locations show a better sinusoidal pattern.

## 4. Conclusions

In this paper, multiple fiber grating sensing elements are implanted into the FRP plate to construct different scales of damage to the FRP plate, and the strain information of the FRP plate is obtained based on the FBG sensors to investigate the effectiveness of the FBG sensors in identifying the damage of the FRP plate. The test results show that the FBG sensor can effectively identify the damage of the FRP plate, and the strain values are different in different directions at the same position. In addition, the damage will lead to stress redistribution; the same location in different directions of the strain size is not the same; and the strain near the location of the damage may be smaller than the strain far from the damage location, that is to say, the degree of damage and the location of the FRP plate for the distribution of stress have a greater impact, which leads to a position at the strain increment, and the degree of the damage change trend is not the same.

In this paper, when designing the FRP plate damage test, the damage was intentionally set near the fiber Bragg grating sensor. In actual working conditions, FRP plate damage is somewhat random; for this reason, continuous strain field monitoring of FRP plates can be carried out by using BOTDR technology and OFDR technology, which can more effectively improve the monitoring effect of FRP plates.

## Figures and Tables

**Table 1:** The initial wavelength of FBGs (nm).

FBG1	FBG2	FBG3	FBG4	FBG5	FBG6
1532.1700	1546.6410	1550.2640	1560.4582	1541.3462	1535.4489

## Data Availability

The original contributions presented in the study are included in the article, further inquiries can be directed to the corresponding author.

## References

[1] Bilotta A., Compagnone A., Esposito L., Nigro E. (2020). Structural behavior of FRP reinforced concrete slabs in fire. Eng. Struct..

[2] Kharghani N., Soares C.G. (2018). Experimental and numerical study of hybrid steel-FRP balcony overhang of ships under shear and bending. Mar. Struct..

[3] Sun Z., Zheng Y., Sun Y., Shao X., Wu G. (2023). Deformation ability of precast concrete columns reinforced with steel-FRP composite bars (SFCBs) based on the DIC method. J. Build. Eng..

[4] Liu C., Wang X., Chang X., Wu Z., Huang H., Noori M., Altabey W.A. (2024). Innovative design and sensing performance of a novel large-strain sensor for prestressed FRP plates. Dev. Built Environ..

[5] Kozioł M., Toroń B., Szperlich P., Jesionek M. (2019). Fabrication of a piezoelectric strain sensor based on SbSI nanowires as a structural element of a FRP laminate. Compos. Part B Eng..

[6] Su Y., Xu L., Zhou P., Yang J., Wang K., Zhou L.-M., Su Z. (2022). In situ cure monitoring and In-service impact localization of FRPs using Pre-implanted nanocomposite sensors. Compos. Part A Appl. Sci. Manuf..

[7] Armonico A., Ferrier E., Michel L. (2024). Smart monitoring of RC T beams strengthened by external bonded FRP. Procedia Struct. Integr..

[8] Zhang Z., Zhou Z., He J. (2025). Development of FBG-based road ice thickness monitoring sensor and its application on the traffic road. Opt. Fiber Technol..

[9] Xue Y., He J., Zhang D., Liu W. (2023). Development of OF based Intelligent Geotextile and Its Case Study in High-speed Railway Subgrade. Measurement.

[10] Wang Q., Zhao K., Badar M., Yi X., Lu P., Buric M., Mao Z.-H., Chen K.P. (2022). Improving OFDR distributed fiber sensing by fibers with enhanced Rayleigh backscattering and image processing. IEEE Sens. J..

[11] Zhao J., Dong W., Hinds T., Li Y., Splain Z., Zhong S., Wang Q., Bajaj N., To A., Ahmed M. (2023). Embedded fiber Bragg grating (FBG) sensors fabricated by ultrasonic additive manufacturing for high-frequency dynamic strain measurements. IEEE Sens. J..

[12] Huang M., Zhou Z., Huang Y., Ou J. (2013). A distributed self-sensing FRP anchor rod with built-in optical fiber sensor. Measurement.

[13] Chen C., Zhang C., Ma J., He S.-Z., Chen J., Sun L., Wang H.-P. (2024). FBG Sensing Data Motivated Dynamic Feature Assessment of the Complicated CFRP Antenna Beam under Various Vibration Modes. Buildings.

[14] Shi J., Kong D., Li C., Guo R., Xian G., Liu H., Tan X. (2025). Strain and damage monitoring of optical fiber sensor-embedded carbon fiber reinforced polymer confined cracked pipes. Polym. Compos..

[15] Zhou Z., He J., Yan K., Ou J. (2008). Fiber-Reinforced polymer-packaged optical fiber sensors based on Brillouin optical time-domain analysis. Opt. Eng..

[16] Wang H., Dai J.-G. (2019). Strain transfer analysis of fiber Bragg grating sensor assembled composite structures subjected to thermal loading. Compos. Part B Eng..

[17] Wang H., Jiang L., Xiang P. (2018). Improving the durability of the optical fiber sensor based on strain transfer analysis. Opt. Fiber Technol..

[18] Lau K.-T., Yuan L., Zhou L.-M., Wu J., Woo C.-H. (2001). Strain monitoring in FRP laminates and concrete beams using FBG sensors. Compos. Struct..

[19] Liu T., Fu Y., Li K., Zhou A., Qin R., Zou D. (2025). Experimental investigation and theoretical analysis of long-term performance for optical fiber Bragg grating-fiber reinforced composite in alkaline concrete environment. Case Stud. Constr. Mater..

[20] Siwowski T., Rajchel M., Howiacki T., Sieńko R., Bednarski Ł. (2021). Distributed fibre optic sensors in FRP composite bridge monitoring: Validation through proof load tests. Eng. Struct..

[21] Anastasopoulos D., Reynders E.P., François S., De Roeck G., Van Lysebetten G., Van Itterbeeck P., Huybrechts N. (2022). Vibration-based monitoring of an FRP footbridge with embedded fiber-Bragg gratings: Influence of temperature vs. damage. Compos. Struct..

[22] Kocaman E.S., Akay E., Yilmaz C., Turkmen H.S., Misirlioglu I.B., Suleman A., Yildiz M. (2017). Monitoring the damage state of fiber reinforced composites using an FBG network for failure prediction. Materials.

[23] Yashiro S., Takeda N., Okabe T., Sekine H. (2005). A new approach to predicting multiple damage states in composite laminates with embedded FBG sensors. Compos. Sci. Technol..

[24] Li R., Tan Y., Chen Y., Hong L., Zhou Z. (2019). Investigation of sensitivity enhancing and temperature compensation for fiber Bragg grating (FBG)-based strain sensor. OFT.

[25] Hill K.O., Malo B., Bilodeau F., Johnson D.C., Albert J. (1993). Bragg grating fabricated in monomode photosensitive optical fiber by UV exposure through a phase mask. Appl. Phys. Lett..

